# Feature selection and risk prediction for diabetic patients with ketoacidosis based on MIMIC-IV

**DOI:** 10.3389/fendo.2024.1344277

**Published:** 2024-03-27

**Authors:** Yang Liu, Wei Mo, He Wang, Zixin Shao, Yanping Zeng, Jianlu Bi

**Affiliations:** ^1^ Endocrinology, The Fifth Clinical College of Guangzhou University of Chinese Medicine, Guangzhou, China; ^2^ Endocrinology, Guangdong Provincial Second Hospital of Traditional Chinese Medicine, Guangzhou, China

**Keywords:** diabetic ketoacidosis, diabetes mellitus, MIMIC-IV, feature selection, risk prediction

## Abstract

**Background:**

Diabetic ketoacidosis (DKA) is a frequent acute complication of diabetes mellitus (DM). It develops quickly, produces severe symptoms, and greatly affects the lives and health of individuals with DM.This article utilizes machine learning methods to examine the baseline characteristics that significantly contribute to the development of DKA. Its goal is to identify and prevent DKA in a targeted and early manner.

**Methods:**

This study selected 2382 eligible diabetic patients from the MIMIC-IV dataset, including 1193 DM patients with ketoacidosis and 1186 DM patients without ketoacidosis. A total of 42 baseline characteristics were included in this research. The research process was as follows: Firstly, important features were selected through Pearson correlation analysis and random forest to identify the relevant physiological indicators associated with DKA. Next, logistic regression was used to individually predict DKA based on the 42 baseline characteristics, analyzing the impact of different physiological indicators on the experimental results. Finally, the prediction of ketoacidosis was performed by combining feature selection with machine learning models include logistic regression, XGBoost, decision tree, random forest, support vector machine, and k-nearest neighbors classifier.

**Results:**

Based on the importance analysis conducted using different feature selection methods, the top five features in terms of importance were identified as mean hematocrit (haematocrit_mean), mean hemoglobin (haemoglobin_mean), mean anion gap (aniongap_mean), age, and Charlson comorbidity index (charlson_comorbidity_index). These features were found to have significant relevance in predicting DKA. In the individual prediction using logistic regression, these five features have been proven to be effective, with F1 scores of 1.000 for hematocrit mean, 0.978 for haemoglobin_mean, 0.747 for age, 0.692 for aniongap_mean and 0.666 for charlson_comorbidity_index. These F1 scores indicate the effectiveness of each feature in predicting DKA, with the highest score achieved by mean hematocrit. In the prediction of DKA using machine learning models, including logistic regression, XGBoost, decision tree, and random forest demonstrated excellent results, achieving an F1 score of 1.000. Additionally, by applying feature selection techniques, noticeable improvements were observed in the experimental performance of the support vector machine and k-nearest neighbors classifier.

**Conclusion:**

The study found that hematocrit, hemoglobin, anion gap, age, and Charlson comorbidity index are closely associated with ketoacidosis. In clinical practice, these five baseline characteristics should be given with the special attention to achieve early detection and treatment, thus reducing the incidence of the disease.

## 1 Introduction

Diabetic ketoacidosis (DKA) is a potentially life-threatening metabolic complication associated with diabetes mellitus (DM). DKA is characterized by a severe lack of insulin and increased levels of counter-regulatory hormones, which can cause the accumulation of ketones in the body. If not promptly diagnosed and treated, DKA can lead to serious complications and even death. Therefore, it is critical to closely monitor DM and take appropriate measures to prevent DKA from developing or to swiftly manage it (1). DKA can develop rapidly, often taking place within 24 hours (2). It can even occur earlier in patients treated with short-acting insulin, such as Humalog, with metabolic changes potentially occurring 1.5 to 2 hours sooner (3). Infection is a frequent precipitating factor for DKA worldwide and accounts for approximately 30-50% of DKA cases. Among potential infections, urinary tract infections and pneumonia are among the most commonly associated with DKA. Other factors that can trigger DKA include concurrent health conditions such as surgical procedures, trauma, myocardial ischemia, and pancreatitis. Psychological stress and medication non-compliance, particularly with insulin therapy, can also contribute to the development of DKA (4).

One of the main triggers for DKA is insufficient insulin. In the absence of adequate insulin, blood glucose levels rise, leading to increased breakdown of triglycerides in adipose tissue and release of a large amount of free fatty acids. More free fatty acids enter the kidneys through the liver, causing an increase in gluconeogenesis in the liver and releasing more glucose into the bloodstream. In an environment of high blood glucose and insufficient insulin, the liver begins to excessively produce ketone bodies, including *beta*-hydroxybutyric acid, acetoacetate, and acetone. The accumulation of ketone bodies in the blood results in increased blood acidity, ultimately leading to ketoacidosis. Ketoacidosis is one of the most significant physiological effects of DKA. Excessive ketone bodies cause an increase in blood acidity, affecting acid-base balance and potentially leading to an acidotic state. Due to increased urine output caused by high blood glucose and ketoacidosis, patients may experience severe dehydration. This can lead to electrolyte imbalances, reduced blood volume, and blood concentration. Dehydration and hyperglycemia may result in disturbances of sodium, potassium, and other electrolytes, potentially triggering arrhythmias and other severe physiological problems. DKA can negatively impact multiple organs, including the heart, kidneys, and nervous system. Recent progress in medical technology has led to significant advances in treatment options for DM. However, despite these developments, the incidence and mortality rates associated with DKA remain high. As the global prevalence of DM continues to rise, the incidence of DKA is also increasing year by year (5). A study involving 28,770 individuals under the age of 20 with DM found that among these participants, 94% did not experience DKA, 5% had a single episode of DKA, and 1% had at least two episodes of DKA (6). The mortality rate for DKA varies between 1% and 5%, with the highest mortality rates typically observed among elderly individuals and those with complications related to their diabetes (7). It is worth noting that cerebral edema, a complication that can occur as a result of DKA, is the leading cause of death among individuals under the age of 24 with DM (8).

Research has shown that there are 100,000 hospitalization cases of DKA in the United States every year, accounting for 4-9% of all discharge records of diabetic patients (4). The treatment of DKA requires a significant amount of healthcare resources. In adult type 1 diabetes patients in the United States, direct medical care costs account for 1/4 of the total expenses (9). Indeed, effective control and prevention of DKA are paramount in reducing healthcare costs. The emergence of computer technology has opened up new avenues for utilizing machine learning techniques to support doctors in disease diagnosis. By leveraging these technologies, healthcare professionals can potentially enhance their diagnostic accuracy and efficiency, leading to improved patient care and cost-effectiveness. Furthermore, given the high risk and poor prognosis associated with DKA, the development of a risk prediction model specifically for this condition is of great importance. Such a model can aid in identifying patients who are at higher risk of experiencing DKA, allowing for targeted interventions and preventive measures. By implementing a risk prediction model, healthcare providers can potentially reduce the incidence of DKA episodes, improve patient outcomes, and mitigate the economic burden on both the healthcare system and patients (10).

**Figure 1:**
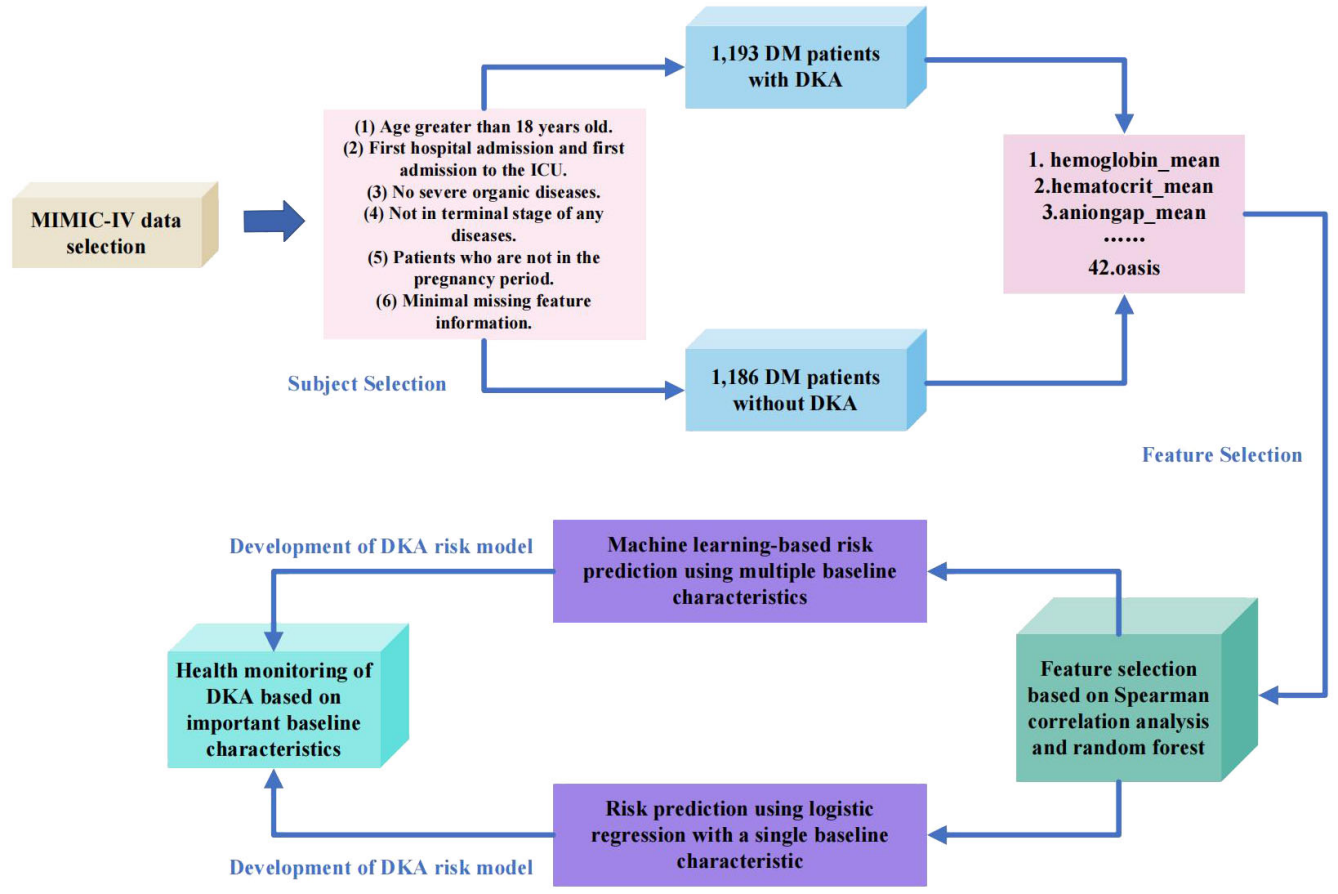
Flow chart of this study.

This study combines the existing public dataset MIMIC-IV with machine learning techniques for healthcare analysis. By employing feature selection methods (random forest, Spearman correlation analysis), baseline characteristics are optimized to identify five baseline characteristics highly correlated with DKA. Based on the abnormality of these five highly correlated baseline characteristics, early warning can be given in the early stages of the disease, assisting clinicians in clinical diagnosis, providing more effective treatment plans, and reducing the incidence of the disease and patients’ suffering. Meanwhile, this study utilizes six machine learning methods to establish a risk prediction model based on DKA, including logistic regression, XGBoost, decision tree, random forest, support vector machine, and k-nearest neighbors classifier. Experimental results demonstrate the effectiveness of feature selection, as the five optimized baseline characteristics can accurately predict the risk of DKA. The research process of this paper is as depicted in **Figure 1**.

## 2 Method

### 2.1 Databaset

The MIMIC dataset was established in 2003 with the support of the National Institutes of Health in the United States. It was jointly created by the MIT Laboratory for Computational Physiology, the Beth Israel Deaconess Medical Center (BIDMC) affiliated with Harvard Medical School, and Philips Healthcare (10). The dataset utilized in this study is known as the ‘Medical Information Mart for Intensive Care IV’ (MIMIC-IV). It encompasses a wide range of data, including demographic information, disease diagnoses, vital signs, laboratory tests, treatment details, survival status, and other comprehensive clinical records. Compared to its predecessor, MIMIC-III, the scope of the MIMIC-IV dataset has been extended to cover the period from 2008 to 2019, providing a broader range of data for analysis and research.

### 2.2 Participant selection criteria

In this study, a total of 2379 patients were chosen from the MIMIC-IV dataset. Among them, 1193 patients had DKA and 1186 patients had DM without ketosis. The participants in this study were required to meet the following criteria: The participants in this study needed to meet the following criteria: (1) Age over 18 years. (2) First admission and first admission to the ICU. (3) Absence of other serious organic diseases. (4) Exclude late-stage disease. (5) Non-pregnant patients. (6) Minimal missing characteristic information.

### 2.3 Selection of indicators and data preprocessing

This study excluded baseline characteristics with missing data greater than 30% in MIMIC-IV, such as C-reactive protein, procalcitonin, height, and serum albumin. At the same time, Structured Query Language (SQL) was used to extract data of DKA patients from MIMIC-IV. The baseline characteristics selected in this study included demographic features, vital signs, laboratory indicators, comorbidity indicators, and scoring system indicators. Demographic features included gender, age, weight, and ethnicity. Vital signs included heart rate (heart_rate_mean), respiratory rate (resp_rate_mean), body temperature (temperature_mean), peripheral oxygen saturation (SPO2_mean), systolic blood pressure (SBP_mean), diastolic blood pressure (DBP_mean), and mean blood pressure (mbp_mean). Laboratory indicators included blood urea nitrogen (bun_mean), creatinine (creatinine_mean), urine output, sodium (sodium_mean), potassium (potassium_mean), calcium (calcium_mean), anion gap (anioinga_mean), hematocrit (haematocrit_mean), hemoglobin (haemoglobin_mean), white blood cell count (wbc_mean), absolute neutrophil count (abs_neutrophils_mean), absolute lymphocyte count (abs_lymphocytes_mean), platelets (platelets_mean), mean corpuscular hemoglobin (mch_mean), red blood cells (rbc_mean), red cell distribution width (rdw_mean), glucose (glucose_mean), and chloride (chloride_mean). Comorbidity indicators included hypertension, obesity, myocardial infarction, congestive heart failure, peripheral vascular disease, chronic pulmonary disease, liver disease, and renal disease. Scoring system indicators included lods, charlson, and oasis.

All data were analyzed using IBM SPSS Statistics 25. Two-sided statistical analyses were conducted, and a significance level of p ≤ 0.05 was used for interpretation of statistical significance. Normality was assessed for continuous variables, which were presented as mean ± standard deviation (SD), while categorical data are summarized as counts or percentages. Group comparisons were performed using the chi-square test for categorical variables and analysis of variance, and the Kruskal-Wallis test for continuous variables. The detailed baseline characteristics are shown in **Table 1**.

**Table 1:** Baseline characteristics between DKA and non-DKA group.

Baseline Characteristics	Total (n = 2379)	DKA (n = 1193)	NO-DKA (n = 1186)	Statistic	P Value
Age	58.92 ± 18.80	47.93 ± 17.26	69.97 ± 12.86	t=-35.332	≤ 0.001
Weight	82.36 ± 24.14	76.49 ± 20.79	88.26 ± 25.78	t=-12.255	≤ 0.001
Heart Rate Mean	87.83 ± 15.37	91.85 ± 14.15	83.79 ± 15.48	t=13.250	≤ 0.001
Sbp Mean	122.07 ± 17.05	123.37 ± 17.73	120.76 ± 16.24	t=3.747	≤ 0.001
Dbp Mean	64.59 ± 11.58	67.48 ± 11.93	61.68 ± 10.44	t=12.632	≤ 0.001
Mbp Mean	79.45 ± 11.47	81.34 ± 12.03	77.55 ± 10.55	t=8.174	≤ 0.001
Resp Rate Mean	19.05 ± 3.65	19.10 ± 3.64	19.01 ± 3.65	t=0.592	0.554
Temperature Mean	36.84 ± 0.51	36.87 ± 0.45	36.81 ± 0.56	t=2.900	0.004
Spo2 Mean	97.22 ± 1.90	97.61 ± 1.70	96.83 ± 2.01	t=10.195	≤ 0.001
Bun Mean	28.45 ± 21.56	28.38 ± 22.16	28.51 ± 20.94	t=-0.145	0.885
Creatinine Mean	1.56 ± 1.69	1.73 ± 2.11	1.38 ± 1.11	t=5.092	≤ 0.001
Urineoutput X	2044.34 ± 1469.52	2287.17 ± 1669.55	1800.09 ± 1187.70	t=8.203	≤ 0.001
Hematocrit Mean	54.92 ± 26.50	76.57 ± 20.71	33.16 ± 5.71	t=69.760	≤ 0.001
Hemoglobin Mean	43.87 ± 35.93	76.57 ± 20.71	11.02 ± 1.96	t=108.807	≤ 0.001
Aniongap Mean	17.15 ± 4.92	19.86 ± 4.78	14.43 ± 3.27	t=32.382	≤ 0.001
Calcium Mean	8.41 ± 0.79	8.39 ± 0.76	8.43 ± 0.81	t=-1.079	0.280
Sodium Mean	137.46 ± 5.03	136.77 ± 5.42	138.16 ± 4.51	t=-6.806	≤ 0.001
Potassium Mean	4.38 ± 0.67	4.47 ± 0.71	4.29 ± 0.61	t=6.822	≤ 0.001
Wbc Mean	12.11 ± 6.23	12.59 ± 6.18	11.63 ± 6.26	t=3.781	≤ 0.001
Abs Lymphocytes Mean	112.51 ± 185.07	87.42 ± 82.36	137.75 ± 246.25	t=-6.694	≤ 0.001
Abs Neutrophils Mean	823.62 ± 591.64	694.22 ± 643.94	953.80 ± 501.50	t=-10.973	≤ 0.001
Platelets Mean	239.49 ± 108.61	261.78 ± 107.41	217.06 ± 105.18	t=10.260	≤ 0.001
Mch Mean	29.79 ± 2.56	29.63 ± 2.56	29.95 ± 2.55	t=-3.018	0.003
Rbc Mean	3.77 ± 0.72	3.85 ± 0.75	3.70 ± 0.67	t=5.118	≤ 0.001
Rdw Mean	14.64 ± 2.00	14.24 ± 1.97	15.05 ± 1.95	t=-10.028	≤ 0.001
Glucose Mean	271.95 ± 2783.01	272.15 ± 1536.30	271.75 ± 3628.83	t=0.003	0.997
Chloride Mean	103.29 ± 6.22	102.67 ± 6.75	103.90 ± 5.58	t=-4.850	≤ 0.001
Lods	4.28 ± 2.98	3.83 ± 2.87	4.73 ± 3.01	t=-7.454	<.001
Charlson Comorbidity Index X	5.70 ± 3.05	4.54 ± 3.07	6.87 ± 2.53	t=-20.233	≤ 0.001
Oasis	29.81 ± 9.20	27.35 ± 8.80	32.29 ± 8.93	t=-13.605	≤ 0.001
Gender, n (%)				$\chi^{2}$ =17.387	≤ 0.001
female	1147 (48.21)	626 (52.47)	521 (43.93)		
male	1232 (51.79)	567 (47.53)	665 (56.07)		
Ethnicity, n (%)				–	≤ .001
AMERICAN INDIAN/ALASKA NATIVE	7 (0.29)	2 (0.17)	5 (0.42)		
ASIAN	61 (2.56)	25 (2.10)	36 (3.04)		
BLACK/AFRICAN AMERICAN	466 (19.59)	323 (27.07)	143 (12.06)		
ethnicity	1 (0.04)	0 (0.00)	1 (0.08)		
HISPANIC/LATINO	105 (4.41)	63 (5.28)	42 (3.54)		
OTHER	103 (4.33)	50 (4.19)	53 (4.47)		
UNABLE TO OBTAIN	29 (1.22)	5 (0.42)	24 (2.02)		
UNKNOWN	169 (7.1)	50 (4.19)	119 (10.03)		
WHITE	1438 (60.45)	675 (56.58)	763 (64.33)		
Mechvent, n (%)				$\chi^{2}$ =134.582	≤ 0.001
No	1861 (78.23)	1050 (88.01)	811 (68.38)		
Yes	518 (21.77)	143 (11.99)	375 (31.62)		
Hypertension, n (%)				$\chi^{2}$ =109.433	≤ 0.001
No	1290 (54.22)	774 (64.88)	516 (43.51)		
Yes	1089 (45.78)	419 (35.12)	670 (56.49)		
Obesity, n (%)				$\chi^{2}$ =46.636	≤ 0.001
No	2096 (88.1)	1105 (92.62)	991 (83.56)		
Yes	283 (11.9)	88 (7.38)	195 (16.44)		
Cad, n (%)				$\chi^{2}$ =142.136	≤ 0.001
No	1714 (72.05)	990 (82.98)	724 (61.05)		
Yes	665 (27.95)	203 (17.02)	462 (38.95)		
Myocardial Infarct, n (%)				$\chi^{2}$ =13.257	≤ 0.001
No	1933 (81.25)	1004 (84.16)	929 (78.33)		
Yes	446 (18.75)	189 (15.84)	257 (21.67)		
Congestive Heart Failure, n (%)				$\chi^{2}$ =98.902	≤ 0.001
No	1778 (74.74)	997 (83.57)	781 (65.85)		
Yes	601 (25.26)	196 (16.43)	405 (34.15)		
Peripheral Vascular Disease, n (%)				$\chi^{2}$ =14.902	≤ 0.001
No	2139 (89.91)	1101 (92.29)	1038 (87.52)		
Yes	240 (10.09)	92 (7.71)	148 (12.48)		
Chronic Pulmonary Disease, n (%)				$\chi^{2}$ =88.380	≤ 0.001
No	1818 (76.42)	1009 (84.58)	809 (68.21)		
Yes	561 (23.58)	184 (15.42)	377 (31.79)		
Liver Disease, n (%)				$\chi^{2}$ =115.167	≤ 0.001
No	2001 (84.11)	1078 (90.36)	923 (77.82)		
Yes	378 (15.89)	115 (9.64)	263 (22.18)		
Renal Disease, n (%)				$\chi^{2}$ =217.075	≤ 0.001
No	1893 (79.57)	868 (72.76)	1025 (86.42)		
Yes	486 (20.43)	325 (27.24)	161 (13.58)		

The LODS (Logistic Organ Dysfunction System) is a medical scoring system commonly used to assess the degree of organ dysfunction in patients. This scoring system evaluates and quantifies the functional status of multiple organ systems based on clinical indicators such as blood pressure, respiratory rate, and oxygen saturation to determine the presence of organ dysfunction in patients.

In the field of home healthcare, OASIS (Outcome and Assessment Information Set) commonly refers to an assessment tool used to collect and document clinical information and functional status data of patients in a home care setting. OASIS assessment covers multiple domains, including activities of daily living, medical history, pain assessment, medication management, emotional status, and more.

The Charlson Comorbidity Index is a scoring system used to assess the burden of comorbidities or other chronic medical conditions in a patient. It assigns a score to various comorbidities based on their association with one-year mortality. The scores are summed to calculate a total score, which is used as an indicator of the patient’s overall health status and the risk of future complications or mortality.

Before carrying out feature selection and developing a DKA risk prediction model, we used mean imputation to handle missing values in the data set. Mean imputation is a commonly used method where the missing values are replaced with the mean or mode of the available data. The formula (Equation 1) for mean imputation can be represented as:


(1) $$\bar{y}=\frac{\sum_{i=1}^{n}\beta_{i}y_{i}}{n_{i}}$$

The symbols indicating whether an answer is provided represent the number of samples. In this study, mean imputation was performed for missing values in neutrophil and lymphocyte counts.

### 2.4 Feature selection

The study employed two feature selection methods to screen important baseline characteristics related to DKA, including Spearman correlation analysis and random forest. Spearman correlation analysis is used to assess the monotonic relationship between two continuous or ordinal variables. It is used to describe the correlation between two variables that have ordinal variables or distribution characteristics that cannot be described by mean and standard deviation. The formula (Equation 2) can be represented as:


(2) $$\rho_{x,y}=\frac{\sum_{i=1}^{N}\left(x_{i}-\bar{x}\right)\left(y_{i}-\bar{y}\right)}{{\left[\sum_{i=1}^{N}{\left(x_{i}-\bar{x}\right)}^{2}\sum_{i=1}^{N}{\left(y_{i}-\bar{y}\right)}^{2}\right]}^{\frac{1}{2}}}$$

Where N represents the total number of observations, *ρ* ranges from -1 to 1. [-1, 0) represents a negative correlation, and (0, 1] represents a positive correlation. A correlation of 0.8-1.0 indicates a very strong correlation, 0.6-0.8 indicates a strong correlation, 0.4-0.6 indicates a moderate correlation, 0.2-0.4 indicates a weak correlation, and 0.0-0.2 indicates a very weak or no correlation. It is worth noting that to better reflect the correlation, we took the absolute value of all correlation coefficients. The top 20 baseline characteristics in terms of correlation strength are shown in **Table 2**.

**Table 2:** Top 20 baseline characteristics based on Spearman correlation analysis.

Baseline Characteristics	Relevance	Baseline Characteristics	Relevance
haemoglobin_mean	0.912	obesity	0.140
haematocrit_mean	0.819	potassium_mean	0.139
age	0.586	sodium_mean	0.138
aniongap_mean	0.552	abs_lymphocytes_mean	0.136
charlson_comorbidity_index	0.383	renal_disease	0.134
oasis	0.269	rbc_mean	0.105
heart_rate_mean	0.262	creatinine_mean	0.104
dbp_mean	0.251	chloride_mean	0.099
cad	0.245	gender	0.085
weight	0.243	peripheral_vascular_disease	0.080
mechvent	0.238	liver_disease	0.079
abs_neutrophils_mean	0.219	wbc_mean	0.077
hypertension	0.214	sbp_mean	0.076
platelets_mean	0.206	myocardial_infarct	0.076
spo2_mean	0.205	ethnicity	0.063
congestive_heart_failure	0.204	mch_mean	0.062
rdw_mean	0.201	temperature_mean	0.059
chronic_pulmonary_disease	0.193	calcium_mean	0.021
urineoutput	0.166	resp_rate_mean	0.012
mbp_mean	0.165	bun_mean	0.002
lods	0.151	glucose_mean	0.002

To enhance the reliability of the experimental results, we also incorporated a feature selection method based on random forests. Random forest is a collection classifier composed of multiple decision trees. The classifier ensemble of the random forest is *RF* = {*h*(*X*, *θ_k_*)*,k* = 1,2,3,···*K*}, where *K* is the number of decision trees, and *θ_k_* is a random variable that follows an independent distribution. Under the known conditions of the independent variables, all classifiers are weighted to obtain the optimal selection result. We had a total of 10,000 decision trees, with a training set to test set ratio of 8.5:1.5. Random forest performed repeated sampling on the replaced dataset to obtain 10,000 data subsets, and each subset generate a corresponding decision tree, ultimately forming the DKA important baseline characteristics ensemble. The importance of random forest in selecting relevant indicators is shown in **Table 3**.

**Table 3:** Top 20 baseline characteristics based on feature selection method using random forest.

Baseline Characteristics	Relevance	Baseline Characteristics	Relevance
haemoglobin_mean	0.3573	bun_mean	0.0028
haematocrit_mean	0.2903	lods	0.0026
aniongap_mean	0.0659	temperature_mean	0.0026
age	0.601	chloride_mean	0.0025
glucose_mean	0.0371	mbp_mean	0.0023
abs_lymphocytes_mean	0.0349	rbc_mean	0.0023
abs_neutrophils_mean	0.0335	sbp_mean	0.0020
charlson_comorbidity_index	0.0223	resp_rate_mean	0.0020
weight	0.0089	urineoutput	0.0020
heart_rate_mean	0.0079	mch_mean	0.0018
oasis	0.0068	potassium_mean	0.0016
rdw_mean	0.0060	hypertension	0.0015
platelets_mean	0.0056	renal_disease	0.0015
dbp_mean	0.0055	calcium_mean	0.0015
liver_disease	0.0045	congestive_heart_failure	0.0011
sodium_mean	0.0040	ethnicity	0.0009
mechvent	0.0037	chronic_pulmonary_disease	0.0008
spo2_mean	0.0035	obesity	0.0006
cad	0.0032	gender	0.0003
wbc_mean	0.0029	myocardial_infarct	0.0003
creatinine_mean	0.0029	peripheral_vascular_disease	0.0001

After conducting correlation analysis using two feature selection methods, it was discovered that certain baseline characteristics exhibited high levels of correlation. By combining the importance rankings of baseline characteristics from the two feature selection methods, the top five strongly correlated baseline characteristics were selected based on their smallest sum of importance rankings. These five baseline characteristics include hemoglobin_mean, haematocrit_mean, aniongap_mean, age, and Charlson_comorbidity_index.

### 2.5 Establishing a risk prediction model for DKA

The study utilized supervised machine learning models for the prediction of DKA risk. The experiments were divided into two parts: the first part focused on risk prediction using logistic regression with a single baseline characteristic, while the second part utilized xgboost, decision trees, random forests, support vector machines, and k-nearest neighbors classifiers with multiple baseline characteristics for risk prediction. The complete dataset for the study was divided into training and testing sets, with a ratio of 0.85:0.15. The experiments were then conducted using five-fold cross-validation. The performance evaluation metrics used for the experiments included the area under the curve (AUC) of the receiver operating characteristic (ROC) curve, accuracy, and F1-score. These metrics were utilized to assess the predictive performance of the models, overall accuracy, and the balance between precision and recall in predicting DKA risk.

#### 2.5.1 Risk prediction based on logistic regression with a single baseline characteristic

The study aimed to predict DKA risk independently for each baseline characteristic using logistic regression. Based on **Table 4**, the experimental results were categorized into three levels according to the F1 score: F1 scores higher than 80, F1 scores between 80 and 60, and F1 scores lower than 60. A total of two baseline characteristics, hematocrit mean and hemoglobin_mean, achieved an F1 score greater than 80. There were 20 baseline characteristics (Age, weight, heart_rate_mean, resp_rate_mean, temperature_mean, anioingap,dbp_mean, abs_neutrophils_mean, congestive_heart_failure, platelets_mean, glucose_mean, obesity, myocardial_infarct, peripheral_vascular_disease, chronic_pulmonary_disease, renal_disease, oasis, cad, mechvent, charlson_comorbidity_index) with F1 scores between 60 and 80. The prediction results demonstrated a significant similarity with the feature selection results, highlighting the strong performance of hematocrit mean and hemoglobin_mean compared to other baseline characteristics. This indicated the importance of these two features in predicting DKA risk.

**Table 4:** Characteristic at baseline between DKA and non-DKA group.

Baseline Characteristics	AUC	Acc	Spe	Sen	F1
gender	0.588	0.593	0.544	0.534	0.539
**age**	0.771	0.773	0.740	0.754	0.747
weight	0.631	0.621	0.558	0.723	0.630
ethnicity	0.541	0.571	0.538	0.264	0.354
heart_rate_mean	0.637	0.619	0.549	0.805	0.653
sbp_mean	0.504	0.484	0.448	0.685	0.542
dbp_mean	0.632	0.633	0.582	0.622	0.601
mbp_mean	0.579	0.579	0.526	0.572	0.548
resp_rate_mean	0.503	0.453	0.447	0.962	0.610
temperature_mean	0.622	0.607	0.543	0.754	0.631
spo2_mean	0.561	0.560	0.505	0.572	0.536
bun_mean	0.579	0.582	0.530	0.547	0.538
creatinine_mean	0.501	0.518	0.448	0.352	0.394
urineoutput	0.547	0.557	0.503	0.452	0.476
**haematocrit_mean**	0.980	0.980	0.975	0.981	0.978
**hemoglobin_mean**	1.000	1.000	1.000	1.000	1.000
**aniongap_mean**	0.724	0.728	0.698	0.685	0.692
calcium_mean	0.493	0.487	0.440	0.553	0.490
potassium_mean	0.537	0.549	0.492	0.427	0.457
sodium_mean	0.643	0.649	0.611	0.584	0.598
wbc_mean	0.527	0.529	0.473	0.509	0.490
abs_lymphocytes_mean	0.591	0.605	0.568	0.471	0.515
abs_neutrophils_mean	0.697	0.686	0.613	0.798	0.694
platelets_mean	0.611	0.602	0.541	0.698	0.609
mch_mean	0.545	0.526	0.479	0.717	0.574
rbc_mean	0.525	0.529	0.472	0.490	0.481
rdw_mean	0.593	0.596	0.546	0.559	0.552
glucose_mean	0.698	0.700	0.656	0.685	0.670
chloride_mean	0.564	0.574	0.524	0.471	0.496
mechvent	0.631	0.602	0.531	0.899	0.668
hypertension	0.608	0.605	0.548	0.641	0.591
obesity	0.523	0.481	0.458	0.905	0.608
cad	0.604	0.577	0.515	0.855	0.643
myocardial_infarct	0.530	0.493	0.463	0.874	0.605
congestive_heart_failure	0.596	0.568	0.509	0.849	0.636
peripheral_vascular_disease	0.531	0.487	0.462	0.937	0.619
chronic_pulmonary_disease	0.588	0.560	0.503	0.849	0.632
renal_disease	0.551	0.515	0.476	0.880	0.618
liver_disease	0.563	0.602	0.673	0.207	0.317
lods	0.565	0.551	0.497	0.685	0.576
**charlson_comorbidity_index**	0.714	0.725	0.725	0.616	0.666
oasis	0.666	0.663	0.608	0.685	0.645

#### 2.5.2 Risk prediction based on multiple baseline characteristics using xgboost, decision trees, random forests, support vector machines, and k-nearest neighbors classifiers

To predict DKA, we utilized all 42 baseline characteristics and employed various machine learning algorithms, including xgboost, decision trees, random forests, support vector machines, and k-nearest neighbors classifiers. The specific algorithm parameter details for xgboost were as follows: a learning rate of 0.01, 3000 iterations, a tree depth of 4, and a minimum sum of leaf node sample weight of 5. The decision tree classifier used the Gini coefficient as the splitting criterion and was constructed with a maximum depth of 50. The random forest classifier employs 8 decision trees, each with a maximum depth of 50.The support vector machine classifier used the radial basis function (RBF) kernel. The k-nearest neighbors classifier was configured to use 5 nearest neighbors, and the algorithm for selecting the nearest neighbors was the automatic optimization algorithm available in the scikit-learn library.

The experimental resulted in **Table 5** indicate that xgboost, decision trees, and random forests achieve an AUC, accuracy, and F1-score of 1, which demonstrates their ability to accurately identify DKA patients. However, the performance of the support vector machine and k-nearest neighbors classifiers was comparatively weaker.

**Table 5:** DKA risk prediction based on all baseline characteristics.

Model	AUC	Acc	F1
xgboost	1	1	1
Decision trees	1	1	1
Random forests	1	1	1
Support vector machines	0.800	0.806	0.773
k-nearest neighbors classifiers	0.820	0.815	0.808

We believe that the reason support vector machines and k-nearest neighbors classifiers cannot accurately identify DKA is due to some baseline characteristics interfering with the model’s decision-making. To further predict DKA, we used feature selection to select five baseline characteristics (namely hemoglobin mean, hematocrit mean, aniongap mean, age, and Charlson comorbidity index).

**Figure 2:**
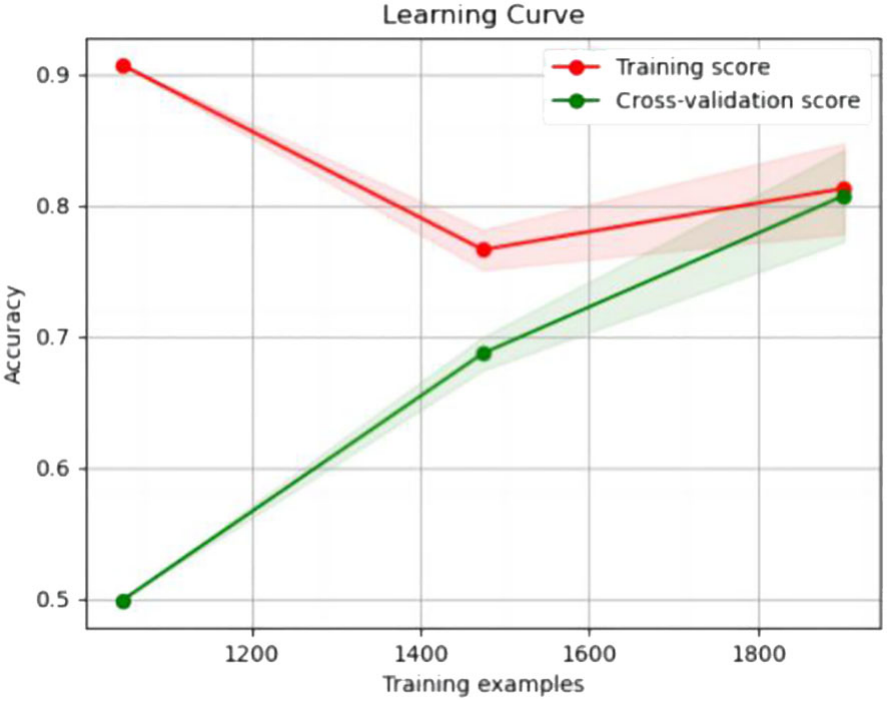
Accuracy change plot of support vector machines based on all features.

**Figure 3:**
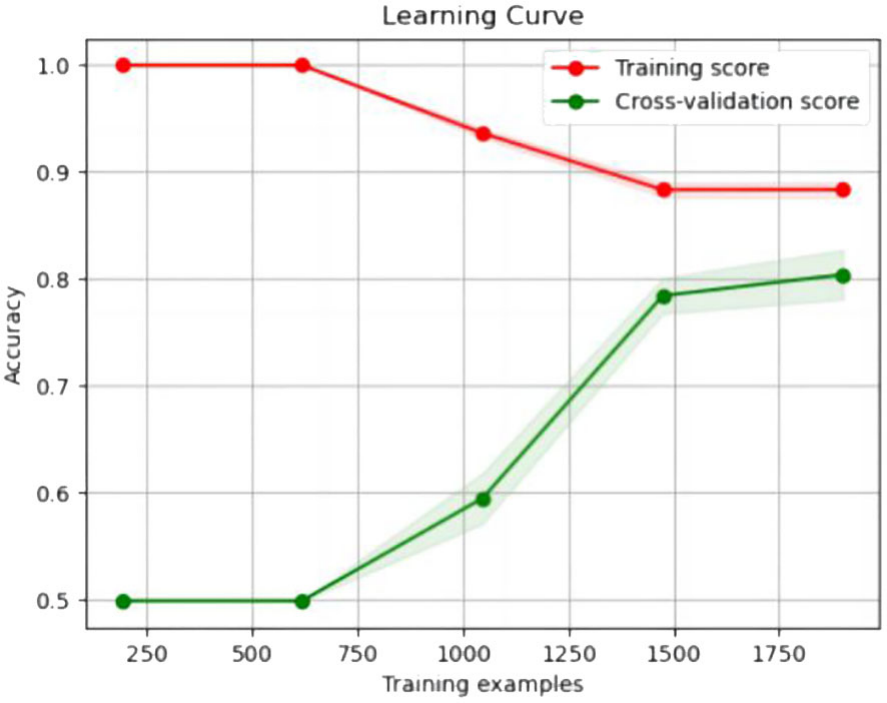
Accuracy change plot of k-nearest neighbors classifier based on all features.

**Figure 4:**
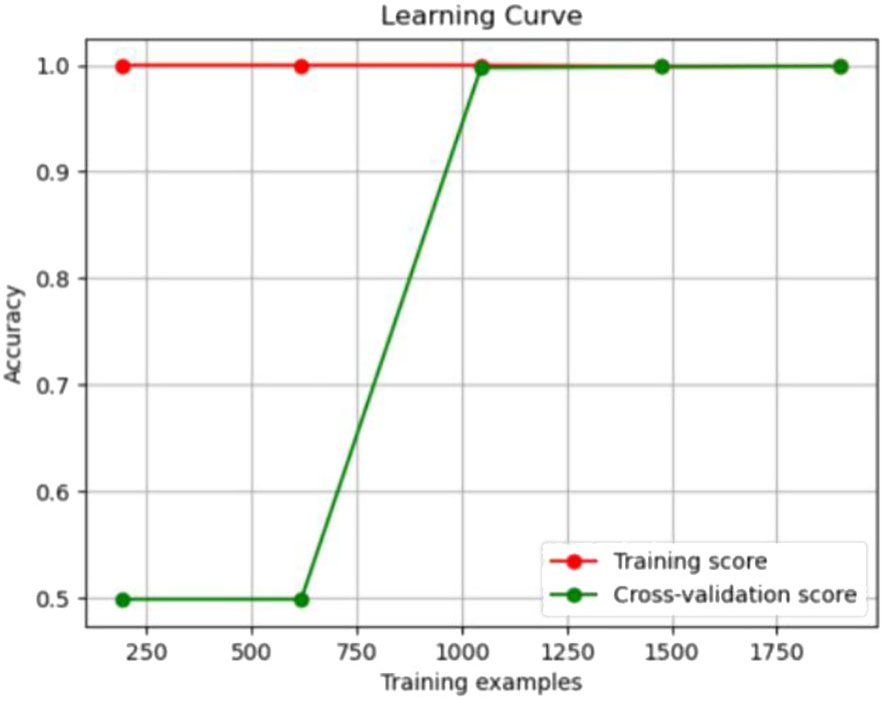
Accuracy change plot of support vector machine based on important features.

**Figure 5:**
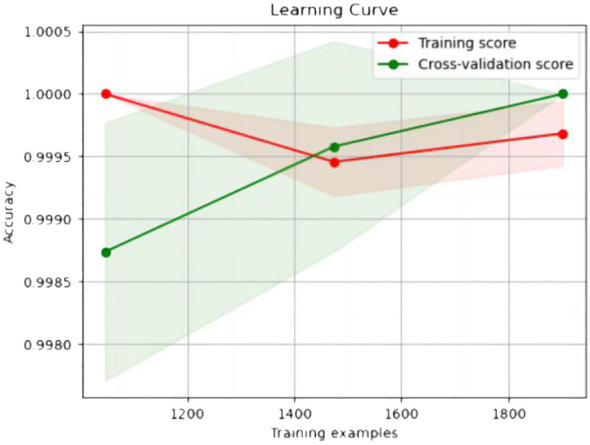
Accuracy change plot of k-nearest neighbors classifier based on important features.

The experimental resulted in **Table 6** demonstrated a significant improvement in the performance of support vector machines and k-nearest neighbors classifiers, validating the effectiveness of the feature selection method and the five important features.We also provided accuracy change plots for support vector machines and k-nearest neighbors classifiers based on both the full set of features and the important features. These plots, labeled as **Figures 2**–**5**, demonstrate the variation in accuracy for the different feature sets. The learning curve illustrated the impact of the number of training samples on the model’s performance. The results indicated that the machine learning approach adopted by the research institute did not exhibit overfitting or underfitting phenomena. The model had essentially reached a performance bottleneck, and there was no need to supplement the data for further training.

**Table 6:** DKA risk prediction based on feature selection.

Model	AUC	Acc	F1
xgboost	1	1	1
Decision trees	1	1	1
Random forests	1	1	1
Support vector machines	1	1	1
k-nearest neighbors classifiers	1	1	1

## 3 Discussion

### Discussion on the importance of baseline characteristics

The occurrence of DKA is attributed to the relative or absolute deficiency of insulin, along with the presence of excessive counter-regulatory hormones such as glucagon, cortisol, catecholamines, and growth hormone. These factors lead to hyperglycemia, glucosuria, dehydration, acidosis, and varying degrees of hyperosmolarity (11). When blood glucose levels elevated, especially in individuals with diabetes, the body is unable to effectively utilize glucose as energy and instead begins to break down fats to provide energy. One of the byproducts of this process is acetoacetic acid. Acetoacetic acid is a ketone body, and when it accumulates excessively in the body, it can lead to ketonemia, which triggers DKA (12). DKA can affect the chemical balance of the blood, including the acid-base balance. It also impacts various parameters related to the blood, such as hemoglobin and hematocrit.

Hemoglobin_mean refers to the mean value of hemoglobin (Hb). Hb is a protein presenting in red blood cells, primarily responsible for carrying and delivering oxygen to various tissues in the body (13). In the state of DKA, due to insufficient insulin or resistance to insulin by cells, is blood glucose levels rise. High blood glucose can lead to excessive urine production by the kidneys, causing significant loss of fluids in the body (14). Inadequate insulin prevents cells from properly utilizing glucose as an energy source. As a result, the body resorts to breaking down fats, leading to an excessive production of ketones in the liver (13). These excess ketones are excreted in urine along with a significant amount of urine, resulting in fluid loss. Glucose is an osmotically active substance, and in a state of high blood glucose, the osmotic pressure of the blood increases, leading to further dehydration of cells. These changes in the body can cause blood to become concentrated, resulting in an increase in the concentration of hemoglobin per unit volume of blood (15). In DKA state, there is a significant increase in acidic substances in the blood. The body utilizes the buffering agents in the blood to neutralize the excess acid, thereby maintaining the acid-base balance of the blood (16). Hemoglobin, a basic protein, can serve as a buffer and increase compensatively in response to acidosis. Thus, changes in HB can effectively reflect the condition of DKA.

Hematocrit_mean represents the mean value of hematocrit (Hct). Hct refers to the proportion of red blood cells in the volume of blood. In clinical practice, Hct is an important indicator for assessing blood concentration and determining blood volume status (17). DKA’s hyperglycemia and ketoacidosis characteristic result in osmotic diuresis and significant depletion of fluid and electrolytes in the intracellular and extracellular fluid compartments (18). The elevated blood glucose and increased urine output caused by DKA lead to dehydration within the body (14) (13). Dehydration-induced blood concentration can cause an increase in Hct. In DKA, the elevated blood glucose and increased concentration of glucose in the blood lead to increased blood viscosity, resulting in an elevated Hct. Therefore, there is a close relationship between Hct and the state changes in DKA.

Hemoglobin and hematocrit are both based on whole blood and therefore depend on plasma volume. If a patient is severely dehydrated, the hemoglobin and hematocrit levels will be higher compared to the normal blood volume (18). An increase in hemoglobin and hematocrit may indicate dehydration and blood concentration (19). Hematocrit and hemoglobin can play a supportive role in evaluating DKA. Given the data from these hematological parameters, such as an increase in red blood cell volume and hemoglobin concentration, they may be useful indicators of inadequate extracellular fluid volume in DKA. Meanwhile, it had been mentioned earlier that cerebral edema was a crucial factor contributing to the increased mortality rate in DKA, primarily due to the most severe complication of excessive or rapid fluid administration. Therefore, accurately assessing the degree of dehydration before initiating fluid therapy in DKA patients was of paramount importance. However, this is not a straightforward estimation, as dehydration did not directly correlate with the severity assessment of DKA based on blood gas values. In this context, hematological parameters can be employed, and two examples were hematocrit (Hct) and hemoglobin (Hb) concentration (10). However, they have limitations in predicting the occurrence of DKA (20), but physiologically, it is reasonable to consider them as useful indicators.

The term ‘anion_gap_mean’ refers to the mean value of anion gap, w hich is used to measure the difference between undetermined anions and undetermined cations in the blood. It is calculated by measuring the concentrations of anions (such as chloride ions) and cations (such as sodium ions, potassium ions) in the blood (21). The formula for anion gap is as follows: Anion Gap = [Na+]-([Cl-] + [HCO3-]) (22). In normal conditions, the anion gap typically falls between 8-16 mmol/L. The anion gap is commonly used to evaluate acid-base balance, and it can be easily calculated from routine laboratory data. It has the widest application in the diagnosis of various forms of metabolic acidosis (23). DKA possesses its unique physiological characteristics, including the generation and elimination of ketones, hyperglycemia, and fluid loss. This combination directly influences the biochemical parameters of patients with DKA, particularly the anion gap and total carbon dioxide levels Mifsud and Salem (11). In the state of DKA, metabolic disturbances in the body lead to the production and accumulation of a large number of ketones, such as beta-hydroxybutyrate, acetoacetate, and acetone. Ketones are metabolic byproducts of fatty acid metabolism, and their breakdown metabolism generates anions, especially beta-hydroxybutyrate. These anions are not accounted for in routine electrolyte analysis and are not included in the sum of cations (such as sodium, potassium) or measured anions (such as chloride) (24). D-lactic acid is a product of methylglyoxal (MG) metabolism through the glyoxalase pathway (25). In a state of hyperglycemia, the production of MG can significantly increase (26). Therefore, in hyperglycemic conditions, the blood concentration of D-lactic acid should also increase significantly. Research has shown that in the state of DKA, the increase in D-lactic acid also contributes to the generation of anion gap during acidosis. Therefore, in DKA, the increase in ketones and D-lactic acid leads to the accumulation of unmeasured anions, r esulting in an increase in the anion gap (24). Therefore, measuring changes in the anion gap can be helpful in diagnosing and monitoring the severity of DKA.

A significant correlation exists between an individual’s age and the likelihood of developing DKA. A study analyzing 4,807 cases of DKA revealed the incidence rate was 14% for those above 70 years old, 23% within the age group of 51 to 70 years, 27% within the age group of 30 to 50 years, and 36% for individuals under 30 years old (5). Based on this data, it is evident that younger patients have a higher incidence rate, with DKA commonly being observed in children and adolescents with both type 1 and type 2 diabetes (27). This is believed to be due to several factors commonly found in patients within this age group, including a higher rate of growth and development, increased metabolic rate, and greater insulin requirements. Furthermore, children and adolescents may have less developed self-management skills for diabetes and may be more susceptible to neglecting or inadequately controlling their blood glucose levels, thus increasing the risk of developing DKA. DKA can affect individuals of all age groups, with older individuals who have additional comorbidities often experiencing higher mortality rates. However, DKA is the leading cause of death among diabetes patients younger than 24 years old, with cerebral edema commonly induced by DKA being the most common cause (8). Middle-aged and elderly patients in this age group may have coexisting chronic conditions such as hypertension, coronary heart disease, and renal failure. These conditions may increase the risk of mortality in DKA and can affect treatment options. Furthermore, elderly patients may have decreased physiological reserves and require careful monitoring of fluid balance and insulin therapy (5). Healthcare providers should develop personalized treatment plans for patients of different age groups, taking into account their physiological characteristics, medical history, and risk of complications. As a result, age plays a crucial role in guiding the management and treatment strategies for DKA. Relevant studies indicate that a mixed state of ketoacidosis and hyperosmolarity is observed in 30% of presentations of hyperglycemic emergencies in diabetes. While both age and the degree of hyperosmolarity influence the mortality rate, only age emerges as an independent predictor of mortality Feldman (12). Poor blood glucose control disproportionately affects young patients with a detrimental impact on DKA. Hence, we emphasize the need for a better understanding of the role of age in diabetes intervention, especially in the context of DKA.

The Charlson Comorbidity Index (CCI), also known as the Charlson Index, is a frequently used instrument for evaluating the burden and risk of comorbidities in patients. It assigns scores to various diseases, depending on a patient’s medical history and diagnoses, and these scores are then combined to generate a composite score (28). CCI offers useful insights into a patient’s overall health status and can assist healthcare professionals in assessing and anticipating the effects of comorbidities on patient outcomes. A high CCI score indicates that the patient is significantly affected by multiple diseases, indicating a greater burden of comorbidities and a higher risk of illness (29). It is widely recognized that many adults with diabetes also experience concurrent chronic conditions such as chronic heart failure, chronic obstructive pulmonary disease, renal disease, and depression (30). In a comprehensive study on medical insurance, it was discovered that the presence of multiple comorbidities can complicate a patient’s condition. The study identified congestive heart failure (CHF), pneumonia (CKD), and chronic obstructive pulmonary disease (COPD) as the most frequent conditions leading to readmission within 30 days after discharge (31). As a result, the proportion of DKA patients with comorbidities such as CHF, CKD, and COPD may be higher, indicating that these conditions commonly coexist in individuals with diabetes, potentially leading to a higher readmission rate for DKA patients. Furthermore, research has suggested that a Hospital Admission Index (HAI) with a CCI score of 3 or higher can serve as a predictive factor for DKA readmission. As previously mentioned, the presence of comorbidities complicates the treatment of diabetes patients, thereby increasing the risk of readmission. Thus, active monitoring and treatment of DKA patients with comorbidities can contribute to enhancing DKA management (32).

The diagnosis of DKA itself is prone to misdiagnosis, and the indicators used are often influenced by the underlying diabetes, making early prediction challenging. The five features we have selected exhibit strong stability, contributing to a comprehensive assessment of the patient’s overall physiological status, not just the diabetes-related physiological changes. In the prodromal stage of DKA, when the values of blood glucose and ketone bodies have not reached diagnostic thresholds, we can complementarily analyze the five features to achieve a comprehensive analysis and provide assistance in predicting DKA. Our intention is not to replace the diagnostic indicators for DKA but rather to serve as an auxiliary indicator to help doctors diagnose more quickly and accurately.

For young patients or those with multiple complications, it is crucial to provide enhanced education and guidance on insulin or medication therapy (33). During the diagnostic and treatment process, it is essential to promptly monitor indicators such as hemoglobin, hematocrit, anion gap, age, and Charlson comorbidity index in DM patients who present with relevant symptoms.Early intervention should be implemented to reduce the incidence of the disease. By closely monitoring these indicators and promptly intervening, the occurrence rate of the disease can be reduced.

## 4 Conclusion

This study was based on the MIMIC-IV dataset and utilized feature selection and machine learning methods to construct a risk prediction model for DKA. Five potential baseline characteristics highly correlated with DKA have been identified, which include hemoglobin_mean, haematocrit_mean, aniongap_mean, age, and Charlson_comorbidity_index. Furthermore, we utilized machine learning methods to accurately predict the incidence of DKA in patients and demonstrated the effectiveness of important baseline characteristics. This study holds the following significant values: (1) Early warning: DKA typically develops gradually rather than occurring suddenly. By continuously monitoring important baseline characteristics and utilizing a machine learning prediction model, it is possible to identify the risk of DM patients progressing to DKA at an early stage, thereby providing early warning signals. This enables doctors to intervene in a timely manner, adjust the patient’s treatment plan, and prevent the occurrence of DKA. (2) Optimization resource allocation: Establishing a DKA risk prediction model can assist hospitals and healthcare institutions in better allocating resources. For instance, for high-risk patients, more attention and resources can be allocated to their monitoring and treatment to reduce the risk of DKA occurrence. This targeted allocation of resources ensures that those at higher risk receive the necessary support and intervention, optimizing the overall healthcare delivery system. (3) Reduction healthcare costs: Treatment for DKA typically requires hospitalization and is associated with high medical expenses. By utilizing important baseline characteristics and predictive models, it is possible to effectively reduce the frequency of DKA episodes, resulting in significant cost savings for patients with recurrent DKA. This cost reduction is achieved through proactive management and prevention strategies based on risk assessment, ultimately improving the overall economic efficiency of healthcare delivery.

There are some limitations associated with this study: (1) Data Quality: The model’s performance heavily relies on the quality of the data used. If there are errors, missing information, or biases in the input data, the model may be influenced by quality variations, impacting its predictive capabilities. (2) Sample Bias: If the samples in the training data are insufficient or do not adequately represent the diversity in the real world, the model may exhibit bias in future practical applications. The representativeness of the samples is crucial for the model’s generalization ability. (3) Concept Drift: If the data distribution changes over time or space, the model may struggle to effectively adapt to the new data distribution. This could result in a decline in the model’s performance in real-world applications. (4) Uncertainty: Machine learning models typically provide probabilities or scores for predictions rather than deterministic outcomes. In the medical field, for certain situations, patients and doctors may prefer to understand the uncertainty of the model rather than just binary predictive results.

## Data availability statement

Publicly available datasets were analyzed in this study. This data can be found here: https://about.citiprogram.org/.

## Ethics statement

The manuscript presents research on animals that do not require ethical approval for their study.

## Author contributions

YL: Writing – original draft, Writing – review & editing, Methodology. WM: Formal analysis, Methodology, Writing – review & editing. HW: Methodology, Writing – review & editing. ZS: Data curation, Investigation, Writing – review & editing. YZ: Data curation, Writing – review & editing. JB: Data curation, Formal analysis, Methodology, Writing – original draft, Writing – review & editing.
